# May mepolizumab used in asthma correct subfertility?

**DOI:** 10.1080/07853890.2021.1900591

**Published:** 2021-03-19

**Authors:** Guzin Ozden, Pelin Pınar Deniz

**Affiliations:** a Department of Internal Medicine and Allergy, Immunology, Adana City Education and Research Hospital, Turkey, Adana; b Department of Chest Medicine, Adana City Education and Research Hospital, Adana, Turkey

**Keywords:** Eosinophilic asthma, mepolizumab, fertility

## Abstract

**İntroduction:**

Asthma is one of the most common chronic airway disease among reproductive period of women. Chronic inflammation in asthma, eosinophilia, high steroid treatment and uncontrolled asthma may cause infertility by affecting the reproductive organs, menstrual cycle and quality of life. Some patients with severe asthma have frequent exacerbations associated with persistent eosinophilic inflammation despite continuous treatment with high-dose inhaled glucocorticoids with or without oral glucocorticoids. Mepolizumab (anti-IL-5) is a succesful option of treatment.

**Cases:**

First case; 25-year-old female patient has been diagnosed having asthma and rhinitis for 5 years. Even she had desired pregnancy for 3 years, she was unable to have a baby, and had been diagnosed having primary infertility. Second case; 36 years old female had rhinitis for 6 years and asthma for 4 years Although she used the same contraception method (withdrawal,condom) for years, she did not get pregnant until receiving the second dose of mepolizumab treatment.

**Result:**

Two women were treated with mepolizumab for eosinophilic severe asthma and they got pregnant.

**Conclusion:**

Unexplained infertility in people with severe eosinophilic asthma may be corrected by mepolizumab treatment. However, there is not enough data regarding the use of mepolizumab during pregnancy.

## Introduction

Asthma is one of the most common chronic inflammatory airway disease among reproductive period of women [1]. The diagnosis of asthma is according to evidence of variable expiratory airflow limitation and history of variable respiratory symptoms (GINA 2019). Severe asthma is a subset of difficult-to-treat asthma that requires treatment with high dose inhaled glucocorticoids plus a second controller (e.g. long-acting beta agonist, leukotriene modifier, theophylline) and/or systemic glucocorticoids for 50% or more of the year [2]. Despite of this treatment, they have also uncontrolled asthma. Mepolizumab (anti-IL-5) is an option of add on therapy in patients with severe eosinophilic asthma [2,3]. It has been shown that the rate of attacks and using steroid are reduced with mepolizumab treatment [4]. Some of previous studies have shown that there mıght be an association with asthma and decreased fertility [5,6]. Chronic inflammation in asthma, eosinophilia, high steroid treatment and uncontrolled asthma may cause infertility by affecting the reproductive organs, menstrual cycle and quality of life.

## Case presentation

### Case 1

A 25-year-old female patient has been diagnosed having asthma and rhinitis for 5 years. She frequently admitted to the emergency room due to dyspnoea and cough increasing in the last 1 year although she received high dose inhaled corticosteroid (ICS), long acting bronchodilation (LABA), theophilline, montelukast, anti-histamine drugs and nasal steroid. Even adding 32 mg of steroid treatment, eosinophil level, FEV1 (forced expiratory volume), PEF (peak expiratory force) were 900 cell/mcL, 81%, and 69 lt/min, respectively. Total IgE was 146 IU/ml and the asthma control test (ACT) score was 8. Skin prick test for inhalant allergens and specific IgE for inhalant allergens (mixture of mite, mould and grasses) were negative. She had frequent exacerbations requiring emergency department visit despite regular use of high dose ICS, LABA, montelukast, anti-histamine drugs and oral corticosteroids (OCS). She had no smoking history. High resolution computed tomography showed no tumoral, infectious or vasculitic abnormality. Even she had desired pregnancy for 3 years, she was unable to have a baby, and had been diagnosed having primary infertility without any identifiable causes other than asthma. Although she took progesterone and clomifen citrate, no pregnancy was occurred. Planned IVF (*in vitro* fertilization) treatment was postponed because of severe asthma attacks. Mepolizumab was started, considering the patient as having severe asthma. After first dose of mepolizumab, eosinophil level, PEF and FEV1 were 100 cell/mcL (1.2%), 73 lt/min, 73% respectively. Asthma control test score was 24. No side effects were seen. Before the third dose of mepolizumab treatment, she had pregnancy of 5 weeks gestational age. She decided to give birth despite the difficulty of the situation. Mepolizumab treatment was ended because there are insufficient data regarding the use of mepolizumab during pregnancy. She started to receive 32 mg of prednisone treatment at the end of 12 weeks. Even receiving high dose steroid and LABA treatment, she had 2 severe attacks during her pregnancy. Due to increased severity of asthma (ACT 8), dyspnoea, cough and eosinophil level of 1800 cell/mcL (24%), she restarted to receive mepolizumab treatment after giving birth. Her dyspnoea was improved, and ACT score is increased to 21 although cessation of steroid treatment by herself. The pregnancy was ended up with a healthy baby without any congenital anomalies.

### Case 2

A 36-year-old married woman with three children did not have any pregnancy. She had rhinitis for 6 years, asthma for 4 years and smoking history. Skin prick test for inhalant allergens and specific IgE for inhalant allergens (mixture of mite, mould and grasses) were negative. High resolution computed tomography showed mosaic parenchymal pattern and pleuroparenchymal sequela changes were detected in the apex of the left lung. A few millimetre sized nodules were detected in the right and left lung. Anti-neutrophilic cytoplasmic antibody (ANCA), anti-nuclear antibody (ANA) results were negative. Eosinophil level, total IgE, FEV1, PEF and ACT were 600 cell/mcL (6.7%), 32 IU/ml, 53%, 49 lt/min and 12, respectively. We started mepolizumab treatment, and after the first dose, eosinophil level was decreased to 100 cell/mcL (1.4%), and ACT score, FEV1 and PEF were improved to 20, 78.8% and 77 lt/min, respectively. Although she used the same contraception method (withdrawal,condom) for years, she did not get pregnant until receiving the second dose of mepolizumab treatment. She decided to terminate pregnancy because of unknown effects of mepolizumab on the foetus.

## Discussion

Asthma is one of the most common chronic inflammatory airway disease among reproductive period in women [1]. Although there are contradictory results in different studies, asthma is generally considered to be a disease resulting in decreased fertility [7–9]. In addition, time to pregnancy has been shown to be decreased by asthma treatment which supports the hypothesis of reduced fertility in asthma [10]. There are a few hypotheses for the explanation of decreased fertility in asthma including asthma related chronic inflammation, chronic use of OCS and stress induced by frequent exacerbations of asthma and decreased quality of life.

Our patients with severe eosinophilic asthma became pregnant after receiving mepolizumab treatment with normal eosinophil count. This may be due to the effect of mepolizumab on eosinophils and asthma control. Eosinophil count is known to be associated with asthma related inflammation and the severity of asthma [11].

Asthma may cause inflammation in other systems including reproductive system through chronic Th 2 (T helper, eosinophilic) inflammation [12]. Gade et al. [13] have been suggested that endometrium might have inflammatory changes. They showed that vascular endothelial growth factor (VEGF) levels decreased in uterine endometrial secretions in asthmatic patients. This decrease in VEGF may result in prolongation of time to pregnancy.

Asthma may change sex steroid hormone levels and cause irregular menses [14]. Another hypothesis of infertility in severe asthma is chronic use of OCS. It is known that exogenous glucocorticoid treatment affects the hypothalamo-pituitary- gonadal (HPG) axis and suppresses the reproductive function. This effect may be due to direct influence of glucocorticoids on hypothalamic receptors or gonads [15–17]. It has been shown that mepolizumab has OCS sparing effect by reducing exacerbations and improving the symptoms of astma [4]. In our patients, mepolizumab improved the symptoms of asthma, and the patients did not need any further OCS treatment. This effect may be the cause of conception early after the first doses of mepolizumab treatment.

Related to glucocorticoid hypothesis, stress induced increased serum endogenous glucocorticoid concentration may cause reproductive dysfunction. Several stress sources such as infection, anxiety and depression elevate serum glucocorticoid concentration and lead to reproductive dysfunction [18]. Severe asthma is also a stress source by decreasing quality of life with frequent exacerbations requiring emergency admission. Mepolizumab has been shown to have beneficial effects on asthma control and improve quality of life [4]. Improved ACT score after mepolizumab treatment in our patients may indicate the improvement in quality of life and reduction in stress. This may result in reduction in endogenous corticosteroids.

## Conclusion

Although the underlying mechanisms of asthma on infertility are largely unknown, these may be endometrial inflammation, OCS treatment or increased endogenous corticosteroids due to stress. Unexplained infertility in people with severe eosinophilic asthma may be corrected by mepolizumab treatment. However, there is not enough data regarding the use of mepolizumab during pregnancy.
